# Supplementary material for: Experiences with using a mobile application for learning evidence-based practice in health and social care education: An interpretive descriptive study
Source: PLoS One. 2021 Jul 12;16(7):e0254272. doi: 10.1371/journal.pone.0254272 (PMC8274852; doi:10.1371/journal.pone.0254272)
Supplement: S1 Appendix — (DOCX) [file pone.0254272.s001.docx]

| **Introduction**  Short introduction to the *EBPsteps* app, with a repetition of its contents and functions. |
| --- |
| **Questions** (ask the bullet points if they do not appear natural during the conversation)   1. Can you describe situations where the *EBPsteps* app was used during clinical placement?  - In which situations (where/when) did you use the app? - Why did you choose to use/not use the app? - Can you describe how the app was used (completed all the steps, to learn, to document clinical placement etc.)? - Can you describe challenges you faced? - Did you experience a need for supervision? Did you get supervision? From whom?  1. How was the *EBPsteps* app useful for your clinical placement?  - Were you able to complete what you wanted when using the app? (Did you find the information you needed?) - In which way was it useful? - What is needed for you to use the app when you start working?  1. How do you work in an evidence-based manner without the *EBPsteps* app?  - How do you ask questions (core questions)? (Documenting questions to find research evidence) - Where/how do you look for research evidence? - How do you critically appraise research evidence/use research?  1. How did you experience the design of the *EBPsteps* app (functionality/technical)?  - What was your first impression of the app? / Was how to use it intuitive? (interfaces, icons) - Was the content recognizable? (EBP, the steps, familiar) - What did you appreciate about the app? Why? - Was there anything you did not appreciate (would like to improve) about the app? Why? - How was it to fill in the questions (easy/difficult, and the same next time)? (manipulation) - How was it to navigate from question to question (easy/difficult)? (navigation) - How easy/difficult was it to understand that what you wrote was automatically stored?  1. How did you experience questions in the *EBPsteps* app (content)?  - We will choose some questions to see if the question formulation works, and ask questions such as: What do you think this question is asking? Can you repeat the question in your own words? What kind of thoughts did you get when you heard this sentence? Can you explain how you chose to answer this question? - Are there terms/expressions/answer alternatives you do not understand? - Are there questions you must read several times to understand? - Are there answers alternatives that are challenging to understand/use? - Are there questions that seem unsuitable/hard to read? |
| **Conclusion**  Are there any additional comments anyone would like to add? |
