# Supplementary material for: Experiences with using a mobile application for learning evidence-based practice in health and social care education: An interpretive descriptive study
Source: PLoS One. 2021 Jul 12;16(7):e0254272. doi: 10.1371/journal.pone.0254272 (PMC8274852; doi:10.1371/journal.pone.0254272)
Supplement: Attachment — Submitted filename: Response to Reviewers.docx [file pone.0254272.s002.docx]

Response to Reviewers	28^th^ of April 2021

Gwo-Jen Hwang
Academic Editor
PLOS ONE

Dear Dr., Gwo-Jen Hwang

Thank you for the opportunity to submit a revised version of our manuscript. We appreciate the constructive criticism and helpful feedback from all of you. A response to each point is provided below. When editing, we also saw the need to make some other minor changes (e.g., spelling). The article will be uploaded with track changes, making it easy to see its modifications.

PONE-D-20-40199
Experiences with using a mobile application for learning evidence-based practice in health and social care education: An interpretive descriptive study
PLOS ONE

Journal requirements:

When submitting your revision, we need you to address these additional requirements.

1. Please ensure that your manuscript meets PLOS ONE's style requirements, including those for file naming. The PLOS ONE style templates can be found at

<https://journals.plos.org/plosone/s/file?id=wjVg/PLOSOne_formatting_sample_main_body.pdf> and

[https://journals.plos.org/plosone/s/file?id=ba62/PLOSOne_formatting_sample_title_authors_affiliations.pdf](about:blank)

**Response:** We have read PLOS ONE's style requirements and updated them to be in line with the journal's requirements.

2. We note that you have indicated that data from this study are available upon request. PLOS only allows data to be available upon request if there are legal or ethical restrictions on sharing data publicly. For information on unacceptable data access restrictions, please see <http://journals.plos.org/plosone/s/data-availability#loc-unacceptable-data-access-restrictions>.

In your revised cover letter, please address the following prompts:

a) If there are ethical or legal restrictions on sharing a de-identified data set, please explain them in detail (e.g., data contain potentially identifying or sensitive patient information) and who has imposed them (e.g., an ethics committee). Please also provide contact information for a data access committee, ethics committee, or other institutional body to which data requests may be sent.

b) If there are no restrictions, please upload the minimal anonymized data set necessary to replicate your study findings as either Supporting Information files or to a stable, public repository and provide us with the relevant URLs, DOIs, or accession numbers. Please see <http://www.bmj.com/content/340/bmj.c181.long> for guidelines on how to de-identify and prepare clinical data for publication. For a list of acceptable repositories, please see <http://journals.plos.org/plosone/s/data-availability#loc-recommended-repositories>.

We will update your Data Availability statement on your behalf to reflect the information you provide.

**Response:** For privacy reasons, it is impossible to provide access to interview data from this project. We did not ask participants for approval for sharing the data. It is also challenging to anonymize the participants in the transcripts. Therefore, due to legal restrictions on sharing the data, it is impossible to share this data.

**Comments to the Author**

1. Is the manuscript technically sound, and do the data support the conclusions?

The manuscript must describe a technically sound piece of scientific research with data that supports the conclusions. Experiments must have been conducted rigorously, with appropriate controls, replication, and sample sizes. The conclusions must be drawn appropriately based on the data presented.

Reviewer #1: Yes

Reviewer #2: Yes

**Response:** We appreciate this feedback.

2. Has the statistical analysis been performed appropriately and rigorously?

Reviewer #1: No

Reviewer #2: Yes

**Response:** We did not use any statistical analysis in this manuscript since we have used a qualitative method.

3. Have the authors made all data underlying the findings in their manuscript fully available?

The [PLOS Data policy](http://www.plosone.org/static/policies.action#sharing) requires authors to make all data underlying the findings described in their manuscript fully available without restriction, with rare exception (please refer to the Data Availability Statement in the manuscript PDF file). The data should be provided as part of the manuscript or its supporting information, or deposited to a public repository. For example, in addition to summary statistics, the data points behind means, medians and variance measures should be available. If there are restrictions on publicly sharing data—e.g. participant privacy or use of data from a third party—those must be specified.

Reviewer #1: Yes

Reviewer #2: Yes

**Response:** We appreciate this feedback.

4. Is the manuscript presented in an intelligible fashion and written in standard English?

PLOS ONE does not copyedit accepted manuscripts, so the language in submitted articles must be clear, correct, and unambiguous. Any typographical or grammatical errors should be corrected at revision, so please note any specific errors here.

Reviewer #1: Yes

Reviewer #2: Yes

**Response:** We appreciate this feedback.

5. Review Comments to the Author

Please use the space provided to explain your answers to the questions above. You may also include additional comments for the Author, including concerns about dual publication, research ethics, or publication ethics. (Please upload your review as an attachment if it exceeds 20,000 characters)

**Response to reviewer 1**

| **Comments** | **Response** |
| --- | --- |
| Reviewer #1: This paper aims to investigate the impacts of Experiences with using a mobile application for learning evidence-based practice in health and social care education: An interpretive descriptive study. It's quite interesting. I agree with the authors' point that this is an EBP health and social care education issue. However, my overall impression of this work is that it is not ready for publication owing to the following reasons: | We appreciate the effort made by both external reviewers and have done our best to respond to each comment where you point to required changes. We refer to page and line in the revised manuscript (version without track changes). |
| Figure 1, " Sample screen of the EBPsteps application " it would be more clear and contribution add related EBP step by step, for example, five EBP steps, should figure out E screen, B screen, P screen. | We appreciate this comment and have corrected it as suggested, page 5, line 104. |
| Page 4, line 80~82: "EBPsteps was launched in 2015. Student representatives and faculties from various professions, including physiotherapy, nursing, occupational therapy, social education and engineering, were involved in its development." it would be better to cite relevant references instead. | We describe people who participated in the development of the *EBPsteps* app. We do not perceive it as relevant to cite references related to this description, page 5, line 106-108. |
| Page 4, line 82~83: "The EBPsteps app guides users through the five EBP steps." It would be better to add more detail about the five EBP steps and to cite relevant references instead. | We have presented the five EBP steps with references, page 5, line 109-111. |
| Page 4, line 87~89: "The app is freely available and can be accessed via any device through the website <http://www.ebpsteps.no> ." For more reader and contrition to improve EBP, why not chose the global popular "PubMed Mobile" app. | PubMed Mobile app relates mainly to the search and retrieval of research articles (two of the EBP steps). The *EBPsteps* were developed to support the learning process of EBP, following the work process of all five EBP steps. Therefore, it was necessary to create a new app. A more thorough explanation is now presented in the article, page 5, line 108-112. |
| Page 5, line 93~94: "Although technology can facilitate EBP teaching, there is still little research in the area of EBP and mobile apps." But I search more then the references below that had discussed related issues, so suggest you describe more your background about your research aim and why it's important.  Morris, J., & Maynard, V. (2010). Pilot Study to Test the Use of a Mobile Device in the Clinical Setting to Access Evidence‐Based Practice Resources. Worldviews on Evidence‐Based Nursing, 7(4), 205-213. Lam, C. K., Schubert, C. F., & Brown, C. A. (2018). Opportunities and Barriers to Building EBP Skills in the Clinical Setting via Mobile Technology. Carlson, K. W. (2018). Perceptions of an EBP Module Mobile Application by New Graduate Nurses. | We appreciate the suggested studies; they are all relevant. The background has been elaborated, and we have included information from these studies, page 4, line 71-83. |
| Page 5~ 6, table1: Participants "… from three different health and social care programmes: occupational therapy (OT), physiotherapy (PT), and social education (SE). We interviewed participants from different programmes to ensure a diversity of experiences related to using the EBPsteps app. The app was introduced to four cohorts of students: second and third-year SE students, third-year OT students, and third-year PT students (Table 1)." So discrepancy EBP teaching sessions*, authors how to ensure their EBP education equal and interview qualitative validity. | It was relevant to examine the student's experiences of using the app based on different EBP teaching experiences, so a diversity of experiences could be uncovered within the focus groups. This was a strength of this study. The different levels of EBP teaching experience were not something the authors could control, page 6, line 135-138. |
| Page 7, line 93~94: "We used a semi-structured interview guide that reflected the aim of the study and previous." it would be better to cite relevant interview guide references instead and for contribution, suggestion authors add the study your detail interview guide. | We have included literature that inspired the interview guide's development and supplied an explanation of usability attributes included in the interview guide. We have clarified that the S1 file is the attachment of the interview guide, page 8, line 165-169. |
| Page 7, line 149~155: "Microsoft Word (23) was used to support the analysis." It would be better to add detail about the interview analysis. Authors how to get the Microsoft Word results and analysis. | We have elaborated how Microsoft Word was used to support the analysis, page 9, line 183. |
| Discussion section, suggest you add your finding linked with transformative Theory, results sections and discuss with Morris & Maynard (2010) and Carlson (2018) and Lam, Schubert,and Brown (2018). | We appreciate this suggestion. The transformative theory is included both in the introduction, page 3, line 48-57, and in the discussion sections, page 20, line 457-459, and line 466-468.  The suggested literature is also included in the discussion, page 18, line 420-421 + page 19, line 440-441 + page 20, line 455-456. |
| Most of the references are too old. | We have updated the literature review with newer references. We believe the included references are relevant to our article, although we have deleted some of the oldest ones. |

**Response to reviewer 2**

| **Comments** | **Response** |
| --- | --- |
| Reviewer #2: The topic of this research is interesting; however, the paper is not well organized and written. | We appreciate the effort made by both external reviewers and have done our best to respond to each comment where you point to required changes. We refer to page and line in the revised manuscript (version without track changes). |
| 1. There are several subsections with only one or two paragraphs, which should be avoided. | Several subtitles have been removed throughout the manuscript. |
| 2. The authors need to reorganize the paper following the standard structure:  Introduction (i.e., background and motivation of the present study)  Literature Review  Development of a mobile application to support learning EBP  Method (or Research design)  Results  Discussion  Conclusion | We appreciate this comment and have included a literature review to the introduction, pages 3-4, lines 68-99. We also ensured that the other parts were organized as suggested. |
| 3. It is suggested that the authors refer to those studies related to the use of mobile technologies in health care or nursing education to strengthen the background and motivation of the present study. Some recommended references are listed as follows:  Chang, C. Y., Kao, C. H., & Hwang, G. J. (2020). Facilitating students' critical thinking and decision making performances: A flipped classroom for neonatal health care training. Educational Technology & Society, 23(2), 32-46.  Chang, C. Y., Gau, M. L., Tang, K. Y., & Hwang, G. J. (2021). Directions of the 100 most cited nursing student education research: A bibliometric and co-citation network analysis. Nurse Education Today, 96, 104645.  Chang, C. Y., Lai, C. L., & Hwang, G. J. (2018). Trends and research issues of mobile learning studies in nursing education: a review of academic publications from 1971 to 2016. Computers & Education,116, 28-48.  Wu, P. H., Hwang, G. J., Su, L. H., & Huang, Y. M. (2012). A context-aware mobile learning system for supporting cognitive apprenticeships in nursing skills training. Educational Technology & Society, 15(1), 223-236. | We appreciate the suggested studies. We have used systematic reviews (SR) as a higher level of synthesis to inform the background, except with the presentation of mobile apps and EBP teaching. Therefore, we have included the suggested SR from your list of studies, page 4, line 85-87 + page 21, line 478. |
