# Supplementary material for: Experiences with using a mobile application for learning evidence-based practice in health and social care education: An interpretive descriptive study
Source: PLoS One. 2021 Jul 12;16(7):e0254272. doi: 10.1371/journal.pone.0254272 (PMC8274852; doi:10.1371/journal.pone.0254272)
Supplement: Attachment — Submitted filename: Response to Reviewers.docx [file pone.0254272.s003.docx]

Response to Reviewers	18^th^ of June 2021

Gwo-Jen Hwang
Academic Editor
PLOS ONE

Dear Dr., Gwo-Jen Hwang

Thank you for the opportunity to submit a second revised version of our manuscript. We appreciate the feedback from all of you. A response to each point is provided below. The article will be uploaded with track changes, making it easy to see its modifications. The version with track changes is where we point to the page and line for changes.

PONE-D-20-40199R1
Experiences with using a mobile application for learning evidence-based practice in health and social care education: an interpretive descriptive study
PLOS ONE


Journal Requirements:

Please review your reference list to ensure that it is complete and correct. If you have cited papers that have been retracted, please include the rationale for doing so in the manuscript text, or remove these references and replace them with relevant current references. Any changes to the reference list should be mentioned in the rebuttal letter that accompanies your revised manuscript. If you need to cite a retracted article, indicate the article's retracted status in the References list and also include a citation and full reference for the retraction notice.

**Response:** We have reviewed the reference list and made sure that it is complete and correct. None of the cited papers were deleted from the manuscript during this review process. We have not cited papers that have been retracted.

[Note: HTML markup is below. Please do not edit.]

Reviewers' comments:

Reviewer's Responses to Questions

**Comments to the Author**

1. If the authors have adequately addressed your comments raised in a previous round of review and you feel that this manuscript is now acceptable for publication, you may indicate that here to bypass the "Comments to the Author" section, enter your conflict of interest statement in the "Confidential to Editor" section, and submit your "Accept" recommendation.

Reviewer #1: (No Response)

Reviewer #2: All comments have been addressed

**Response:** We appreciate the feedback from reviewer #2 from the previous round of review.

2. Is the manuscript technically sound, and do the data support the conclusions?

The manuscript must describe a technically sound piece of scientific research with data that supports the conclusions. Experiments must have been conducted rigorously, with appropriate controls, replication, and sample sizes. The conclusions must be drawn appropriately based on the data presented.

Reviewer #1: No

Reviewer #2: (No Response)

**Response:** As Reviewer #2 has no response regarding this issue, we believe that Reviewer #2 is of the opinion that the manuscript is technically sound and that the data support our conclusion. We notice that Reviewer #1 response is "no", although there is no detailed information about what is missing. We hope that changes we have made to the manuscript regarding other comments (item 6 in the list "6. Review Comments to the Author") are sufficient to ensure that the manuscript is technically sound and that the data support our conclusion.

3. Has the statistical analysis been performed appropriately and rigorously?

Reviewer #1: No

Reviewer #2: Yes

**Response:** As this is a qualitative study, we did not use any statistical analysis in this manuscript.

4. Have the authors made all data underlying the findings in their manuscript fully available?

The [PLOS Data policy](http://www.plosone.org/static/policies.action#sharing) requires authors to make all data underlying the findings described in their manuscript fully available without restriction, with rare exception (please refer to the Data Availability Statement in the manuscript PDF file). The data should be provided as part of the manuscript or its supporting information, or deposited to a public repository. For example, in addition to summary statistics, the data points behind means, medians and variance measures should be available. If there are restrictions on publicly sharing data—e.g. participant privacy or use of data from a third party—those must be specified.

Reviewer #1: No

Reviewer #2: Yes

**Response:** Participants in our study did not agree to the data material being shared, which means that there is a legal and ethical restriction on publicly sharing our data. In line with recommendations given by the Norwegian Centre for Research Data (project no. 50425), data are stored on a secure server at Western Norway University of Applied Sciences (HVL). The Research Integrity Officer at HVL, Anne-Mette Somby (Anne-Mette.Somby@hvl.no), may be contacted if queries regarding verifiability of data.

5. Is the manuscript presented in an intelligible fashion and written in standard English?

PLOS ONE does not copyedit accepted manuscripts, so the language in submitted articles must be clear, correct, and unambiguous. Any typographical or grammatical errors should be corrected at revision, so please note any specific errors here.

Reviewer #1: Yes

Reviewer #2: Yes

**Response:** We appreciate this feedback.

6. Review Comments to the Author

Please use the space provided to explain your answers to the questions above. You may also include additional comments for the author, including concerns about dual publication, research ethics, or publication ethics. (Please upload your review as an attachment if it exceeds 20,000 characters)

**From Reviewer #1:**

Dear Authors,
Thank you for your revised work, but I suggest some advice below,
1.Page 6, line 124-126, "15 students with a mean age of 26 participated in focus group interviews between February and May 2017. Most participants were female 126 (n=14), and most had completed clinical placements in primary care settings (n=13" The Participants section should be rewording based on your Table 1, Table 1. Characteristics of participants not match, for example, Social Education 1, Number of students in the cohort (N=68), but why age only N=4? please check.
in addition, why only 15 students with a mean age of 26 participated in the focus group get an interview? It's vague.

**Response:** We appreciate this advice from Reviewer #1 and have made the following changes to the manuscript:

- When we first introduce Table 1, we state how many students in the different cohorts were introduced to the app, page 6, lines 136-140 (Table 1, line 1).
- Thirty-three students used the app during clinical placement. How many students from each cohort using the app is presented on page 7, lines 153-155 (Table 1, line 2).
- In total, fifteen students agreed to participate. We have written this clearer in the text, page 7, lines 156-158 (Table 1, line 2).
- We reported the student's age on those who participated in this study.

**From Reviewer #1:**
2. Page 9, Results section, should be rewording too because, from table 2, it's difficult to get the related information "Three integrated themes were generated from the analyses: "triggers for EBP", "barriers to EBP", and "design matters".
Besides abstract and results stated that get three integrated themes "triggers for EBP", "barriers to EBP", and "design matters". But exactly, the result section not only three integrated themes, including Triggers for EBP, Information needs in clinical placement, Academic requirements, Barriers to EBP, Lack of EBP knowledge, Lack of academic requirement, Clinical instructors did not require students to apply EBP, EBP was not a priority, Design matters, Motivating design aspects, and Design factors that hindered the use of the app. Suggestion authors amendments, Meanwhile, need to follow your interview guideline.

**Response:** We appreciate this advice from Reviewer #1. Based on this comment, we realized that some clarification was needed. The aim with table 2 was to illustrate the part of the analysis process that involves comparing and contrasting and developing themes. However, this was not clarified sufficiently in the text. We realize that keeping the table could cause confusion and misunderstandings and illustrating all four phases meaningfully in a table is difficult. Therefore, we decided to delete Table 2 from the text. We can however provide this table as an appendix.

Regarding the comment that "the results section should be rewording". The three themes consist of different explanations for each theme presented in the theme's introduction in the results section. For instance, the theme "triggers for EBP" is explained with "The use of the *EBPsteps* app was influenced by factors such as the need for information experienced in patient situations, course work requiring the use of research evidence, or clinical instructors expecting students to find and use research evidence." Thus, it was different aspects that could trigger EBP and the use of the *EBPsteps* app, e.g., information needs in clinical placement, academic requirements, and clinical instructors expect students to apply EBP.

Regarding the comment that we "need to follow your interview guideline". We are unsure how to interpret this comment, and we did not make any changes. The interview guide was developed to support the interviews of the focus groups, and it was not used as a guide for the analysis. We used an inductive process, where the information from the participants guided the analysis process.

**From Reviewer #1:**

3. The discussion and conclusions section should follow the Results section too.

**Response:** We appreciate this advice from Reviewer #1. We have now clarified the link to the themes that emerged: page 19, lines 425-426, and lines 439-440 + page 20, lines 453-454 and line + page 21, line 474 and lines 485-486.

To ensure that the conclusion aligns with the Results section, we have revised the conclusion with additional explanations related to the Results section, page 23, lines 530-532 and 534-538.

Reviewer #2: (No Response)
